# Effect of Early Cyclosporine Treatment on Survival in Stevens-Johnson Syndrome and Toxic Epidermal Necrolysis

**DOI:** 10.7759/cureus.57862

**Published:** 2024-04-08

**Authors:** Atsushi Senda, Kiyohide Fushimi, Koji Morishita

**Affiliations:** 1 Department of Acute Critical Care and Disaster Medicine, Tokyo Medical and Dental University, Tokyo, JPN; 2 Department of Health Policy and Informatics, Tokyo Medical and Dental University, Tokyo, JPN

**Keywords:** toxic epidermal necrolysis, stevens–johnson syndrome, database, cyclosporine, cohort studies

## Abstract

Introduction: Early cyclosporine administration is a potentially useful treatment in patients with Stevens-Johnson syndrome and toxic epidermal necrolysis (SJS/TEN). However, previous studies have reported conflicting results regarding the survival benefits. Therefore, in this study, we evaluated the survival of patients with SJS/TEN according to whether they received early cyclosporine administration.

Methods: This retrospective cohort study was conducted using a Japanese national administrative claims database. Data on patients admitted to the hospital with SJS/TEN between April 1, 2016, and March 31, 2021, were extracted. Patients with missing data, those discharged within two days of admission, pregnant women, and children aged <16 years were excluded. Patients who received cyclosporine on the day of admission (early cyclosporine group) were compared with those who did not (comparison group). The primary endpoint was in-hospital mortality. Secondary endpoints were 30- and 50-day mortality and length of hospital stay. The effect of early cyclosporine treatment was evaluated after baseline adjustment using doubly robust estimation.

Results: Among 3807 enrolled patients (mean age, 65.5 years; 53.8% women), the early cyclosporine and comparison groups included 115 and 3692 patients, respectively. After adjustment, cyclosporine treatment decreased in-hospital mortality by 6.03% (95% confidence interval (CI), 5.27-6.82%), 30-day mortality by 2.94% (95% CI, 2.43-3.50%), and 50-day mortality by 4.38% (95% CI, 3.70-5.04%), but increased the length of hospital stay by 9.45 days (95% CI, 1.00-20.23 days).

Conclusion: Early cyclosporine administration can improve the survival of patients with SJS/TEN but is associated with a longer hospital stay.

## Introduction

Stevens-Johnson syndrome (SJS), toxic epidermal necrolysis (TEN), and their overlapping form (SJS/TEN) are among the most life-threatening cutaneous diseases. SJS/TEN is characterized by painful skin eruptions, purplish macules, and atypical bullseye-shaped lesions that affect the outer skin and internal mucous membranes [1]. This disease can also affect other organs, including the cardiovascular, respiratory, digestive, and urinary systems, potentially leading to complications and sequelae [2].

Recent advances have provided insights into the pathology of SJS/TEN. The condition is primarily triggered by CD8 T cells [3,4] and mediated by granulysin [5]. Immunomodulatory treatments, including corticosteroids [6,7], intravenous immunoglobulin (IVIG) [8], plasmapheresis [9], and anti-tumor necrosis factor-α monoclonal antibodies [10], have been suggested. However, these treatments have not yet been substantiated by ample evidence [11-17].

Cyclosporine, an inhibitor of calcineurin activity that suppresses the antigen-specific activation of T cells [18,19], has emerged as a potential treatment. Given the pathology of SJS/TEN, this treatment appears promising, and an increasing body of evidence supports the survival benefit of the early administration of cyclosporine [20,21]. Nevertheless, no survival advantage was found reciprocally in a recent study [22], necessitating further research. Thus, the aim of this study was to precisely determine whether the early administration of cyclosporine as a treatment for SJS/TEN offers a survival advantage. A retrospective analysis using a nationwide administrative claims database was conducted to address this issue, as randomized controlled trials for SJS/TEN are impracticable owing to their rarity.

## Materials and methods

Research design

This retrospective cohort study used the Japanese Diagnosis Procedure Combination (DPC) database data. We evaluated the treatment effect of early cyclosporine administration on mortality in patients hospitalized with SJS/TEN. The results were reported according to the Strengthening of Observational Studies in Epidemiology guidelines.

Information sources

The DPC database is a comprehensive system for evaluating medical treatment fees during the acute phase of hospitalization. This database maintains patient demographic data (including sex, age, and weight), admission and discharge dates and status, scores reflecting activities of daily living, and post-admission complications. It also records all dispensed medicines, procedures, and healthcare provided to each patient during hospitalization. The diagnoses are assigned specific codes from the International Classification of Diseases, 10th edition (ICD-10). The DPC database has been described in more detail previously [23].

Research participants

Data were collected from patients admitted with a diagnosis of SJS, TEN, or SJS/TEN (ICD codes: L51.1, L51.2, and L51.3) between April 1, 2016, and March 31, 2021. The dataset used in this study was identical to the previously published dataset [24]. The following patients were excluded: those with missing data for any analyzed variables, those aged <16 years, and pregnant women. Patients who died and those who were discharged alive within two days of admission were also excluded to address the immortal-time bias, as the treatment intensity was evaluated based on the treatment received during this period.

Ethical considerations

This study adhered to the 1964 Declaration of Helsinki principles and its later amendments. The study was approved by the Institutional Review Board of Tokyo Medical and Dental University (approval number: 788). The requirement for individual informed consent was waived because of the retrospective study design and anonymization of patient data.

Exposures and outcomes

Patients who received cyclosporine on the day of admission were included in the early cyclosporine group and compared with those who did not receive early cyclosporine therapy (comparison group). The primary outcome was in-hospital mortality. Secondary outcomes were 30- and 50-day mortality and length of hospital stay.

Statistical analysis

Doubly Robust Estimation

The effectiveness of early cyclosporine treatment was assessed using doubly robust estimation. The standardized mean difference (SMD) was calculated using the following equation:

$SMD\ = \ \frac{1}{N}\sum_{i}^{}{(\frac{T_{i}(Y_{i} - {\widehat{\mu}}_{1}(X_{i}))}{\widehat{P}(X_{i})}\ + \ {\widehat{\mu}}_{1}(X_{i}))\ - \ \frac{1}{N}\sum_{i}^{}{(\frac{(1\ - \ T_{i})(Y_{i} - {\widehat{\mu}}_{0}(X_{i}))}{1\ - \ \widehat{P}(X_{i})}\ + \ {\widehat{\mu}}_{0}(X_{i}))}}$

Where $X_{i}$ represents the covariates, $Y_{i}$ represents the outcomes of individual patients, $T_{i}$ indicates whether an individual patient has received treatment (1: treatment received; 0: treatment not received), $\widehat{P}(X_{i})$​​​​​​ represents the propensity score, ${\widehat{\mu}}_{1}(X_{i})​​​​​​$ is an estimation of E(Y|X,T=1), and ${\widehat{\mu}}_{0}(X_{i})​​​​$ is an estimation of E(Y|X,T=0) of a patient.

Model Construction

Using a logistic regression model, this study estimated E(Y|X, T). This model was constructed from a random sample comprising 70% of the patients. $Y_{i}$ represents the in-hospital, 30-day, and 50-day mortality. Covariates $X_{i}$ were selected based on clinical perspective and included patient age, sex, height, weight, smoking index, Charlson Comorbidity Index, diagnosis of diabetes, heart failure, and acute lung injury, level of alertness, treated gross wound area, mechanical ventilation use, renal replacement therapy, IVIG, adrenaline, noradrenaline, dopamine, dobutamine, and hydrocortisone administration, red blood cell transfusion, surgical treatment, plasmapheresis, whether the patient was admitted to the intensive care unit, and type of treating hospital (academic hospital or other).

Model Validation

The model's performance was validated in the remaining 30% of the cohort using the area under the receiver operating characteristic curve (AUROC). The goodness-of-fit was assessed using the Hosmer-Lemeshow test. Propensity scores outside the 99th percentile were trimmed to avoid extreme weighting, as described previously [25,26]. The confidence interval (CI) was estimated using bootstrapping with 3000 replications. All statistical analyses were performed using Python version 3.8.6 (Centrum voor Wiskunde en Informatica, Amsterdam, the Netherlands).

## Results

Patient characteristics

**Figure 1:**
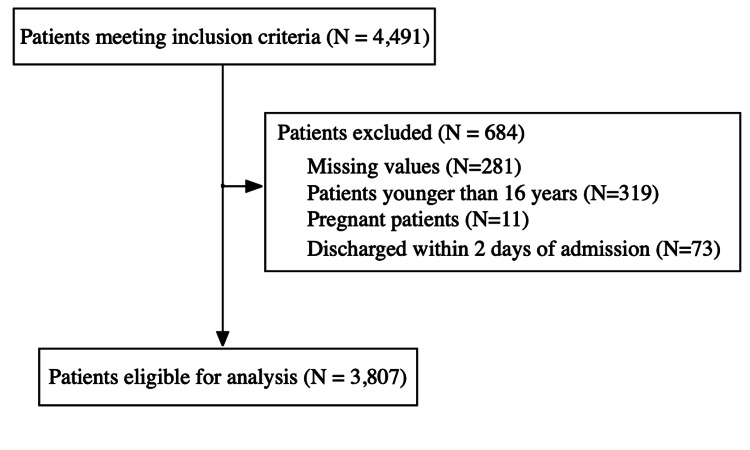
Patient flow diagram

During the study period, 4491 patients were diagnosed with SJS/TEN. Among them, 3807 patients were included in the analysis. The reasons for excluding 684 patients are shown in Figure 1.

Cyclosporine was administered to 115 patients (3.0% of the analyzed population) on the day of admission, and the remaining 3,692 patients did not receive cyclosporine during this period. Patients in the early cyclosporine group tended to be younger, more likely to receive intensive treatment (based on treatment using immunoglobulin, plasmapheresis, and intensive care unit admission), and have fewer complications (including renal replacement therapy, the requirement for mechanical ventilation, and the Charlson Comorbidity Index) than those in the comparison group. The patient characteristics are summarized in Table 1.

**Table 1:** Patient characteristics according to early cyclosporine treatment ICU: intensive care unit, IQR: interquartile range

	Early cyclosporine treatment (N=115)	No early cyclosporine treatment (N=3692)
Age (years), median (IQR)	47 (26–65)	65 (48–76)
Female sex, n (%)	77 (67.0)	1970 (53.4)
Height (cm), median (IQR)	158 (155–165.5)	159 (151–166)
Weight (kg), median (IQR)	49.4 (42.4–59.75)	54.85 (47–64.5)
Consciousness (alert), n (%)	111 (96.5)	3374 (91.4)
Diabetes, n (%)	15 (13.0)	555 (15.0)
Heart failure, n (%)	2 (1.7)	160 (4.3)
Intravenous immunoglobulin use, n (%)	34 (29.6)	735 (19.9)
Hydrocortisone use, n (%)	5 (4.3)	171 (4.6)
Dopamine use, n (%)	1 (0.9)	51 (1.4)
Dobutamine use, n (%)	5 (4.3)	42 (1.1)
Noradrenaline use, n (%)	8 (7.0)	184 (5.0)
Red blood cell transfusion, n (%)	10 (8.7)	293 (7.9)
Plasmapheresis, n (%)	4 (3.5)	35 (0.9)
Renal replacement therapy, n (%)	0 (0)	38 (1.0)
Ventilator use, n (%)	0 (0)	60 (1.6)
Surgical intervention, n (%)	0 (0)	15 (0.4)
ICU admission, n (%)	13 (11.3)	277 (7.5)
Academic hospital, n (%)	59 (51.3)	1269 (34.4)
Acute renal failure, n (%)	2 (1.7)	54 (1.5)
Smoking index, median (IQR)	0 (0–0)	0 (0–600)
Treated gross wound area/day (cm^2^), median (IQR)	3100 (2250–4600)	2750 (2200–4130)
Charlson Comorbidity Index, median (IQR)	0 (0–1)	1 (0–2)

Crude outcomes before disease severity adjustments

The in-hospital, 30-day, and 50-day mortality rates in the comparison group were 5.96%, 2.92%, and 4.36%, respectively. However, no deaths were reported in the early cyclosporine treatment group. The length of hospital stay was longer in the early cyclosporine treatment group (mean, 27 days (interquartile range (IQR), 15-51 days)) than in the comparison group (mean, 18 days (IQR, 11-29 days)).

Development of the model

**Figure 2:**
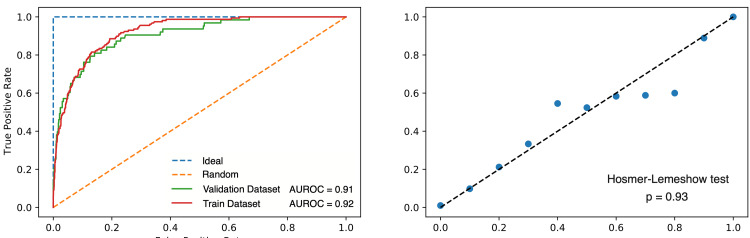
Receiver operating curves and goodness of fit of the case-mix classification model AUROC: area under the receiver operating curve

The validated classification model showed good predictive accuracy and calibration (AUROC = 0.91; Hosmer-Lemeshow goodness-of-fit test: p=0.93; Figure 2).

The incidence rates of acute renal failure (4.35%, 5/115 vs. 4.96%, 183/3692) and sepsis (2.61%, 3/115 vs. 2.38%, 88/3692) did not differ between the early cyclosporine treatment and comparison groups.

Outcomes after disease severity adjustments

The results after adjusting for confounding factors are shown in Table 2.

**Table 2:** Patient characteristics according to early cyclosporine treatment after adjustment for disease severity IQR: interquartile range, ASMD: absolute standard mean difference

	Early cyclosporine treatment	No early cyclosporine treatment	ASMD
Age (years), median (IQR)	61.5 (43.0–79.5)	60.5 (41.5–75.0)	0.02
Female sex, %	49.8	53.8	0.07
Consciousness (alert), %	93	91.5	0.02
Diabetes, %	19.2	15	0.28
Heart failure, %	4	4.3	0.07
Immunoglobulin, %	23.6	20.2	0.17
Hydrocortisone, %	4.6	4.6	0
Dopamine, %	1.4	1.4	0
Dobutamine, %	1.1	1.2	0.08
Noradrenaline, %	3.8	5	0.24
Red blood cell transfusion, %	7.4	7.9	0.06
Plasmapheresis, %	0.7	1	0.3
Renal replacement therapy, %	0	0	0
Ventilator use, %	0	0	0
Surgical intervention, %	0	0	0
Acute renal failure, %	2.2	1.5	0.47
Charlson Comorbidity Index, median (IQR)	1 (0–1)	1 (0–2)	0

**Figure 3:**
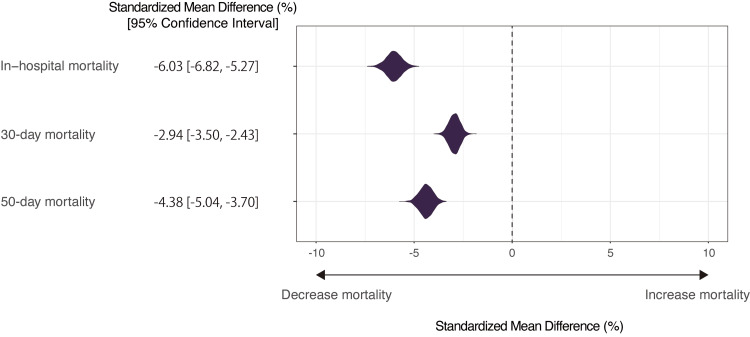
Patient mortality after covariate adjustment comparing patients with and without early cyclosporine treatment

**Figure 4:**
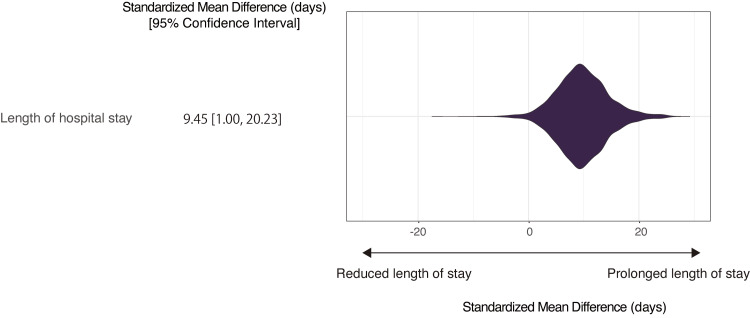
Length of hospital stay after covariate adjustment comparing patients with and without early cyclosporine treatment

After adjustment, the SMDs of the early cyclosporine treatment were as follows: in-hospital mortality (SMD (95% CI), −6.03% (−6.82% to −5.27%)), 30-day mortality (SMD (95% CI), −2.94% (−3.50% to −2.43%)), 50-day mortality (SMD (95% CI), −4.38 (−5.04 to −3.70)), and length of hospital stay (SMD (95% CI), 9.45 days (1.00 to 20.23 days)) (Figures 3-4).

## Discussion

Using a comprehensive national database, this study estimated the effectiveness of early cyclosporine treatment in patients with SJS/TEN. The results indicated a survival benefit owing to early cyclosporine treatment in this patient population. A strength of this study is the large sample size, which is 20 times larger than that of a previous study that included 174 patients with SJS/TEN [22].

To the best of the author's knowledge, no previously reported study has adequately arranged the patient background between the cyclosporine treatment group and the untreated group, as has been done in the present study. Our results are consistent with those of a previous meta-analysis that included 358 patients with SJS/TEN from 12 studies. In the meta-analysis, the pooled standardized mortality ratio was 0.320 (95% CI: 0.119-0.522), indicating a survival benefit of early cyclosporine administration in patients with SJS/TEN [21]. The sample sizes of the studies reviewed in the meta-analysis ranged from three to 44, whereas this study analyzed data on 3,807 patients with SJS/TEN.

In contrast to the findings of this study, another retrospective cohort study reported no survival benefit following the use of cyclosporine [22]. However, careful interpretation is needed, as these findings do not indicate that cyclosporine treatment is futile. The wide CIs (hazard ratio: 0.26-9.28 (propensity score matching) and 0.17-14.6 (multivariable analyses)) suggest an inadequate sample size. Additionally, the patients in this study were selectively allocated. Specifically, 29 patients from the study cohort were included in a phase II trial, with inclusion determined by the trial inclusion criteria [27].

This study showed that early cyclosporine treatment was associated with a prolonged hospital stay. As the incidence rates of acute renal failure and sepsis did not differ between the groups, the prolonged length of hospital stay cannot be explained by the side effects of cyclosporine.

This study had some limitations. Firstly, the verification of diagnostic data in a database might not be as comprehensive as that of data collected for specifically designed prospective studies. However, a previous validation study reported a diagnostic specificity of >96% for the DPC database [28]. Secondly, we excluded patients discharged within two days after admission because the disease severity was estimated based on the intensity of the treatment provided during this period. Thirdly, the possibility of residual confounding cannot be ruled out despite our use of a rigorously validated predictive model owing to the retrospective design of this study and reliance on a database that did not capture all relevant information, such as vital signs and laboratory test results. This may explain the longer length of hospital stay in the early cyclosporine treatment group; the adjustment may have been insufficient, causing an underestimation of the benefit of cyclosporine. Fourthly, the clinical benefit of early treatment with cyclosporine was evaluated. It is noteworthy that the non-early cyclosporine group included patients who received cyclosporine after the defined period. Therefore, the treatment effect of cyclosporine may be underestimated due to Type II errors. Fifthly, in this study, the early cyclosporine treatment group was defined as patients who received cyclosporine on the day of admission. This definition served as a substitute for cases in which cyclosporine therapy was initiated within 24 hours of admission. The substitution was necessitated by restrictions within the database study and should be noted that it potentially leads to Type II errors. Finally, in-hospital mortality may have shortened the average length of hospital stay in the comparison group, leading to survival bias.

## Conclusions

In conclusion, early cyclosporine administration can improve the survival of patients with SJS/TEN. Further studies are warranted to confirm these findings.
